# Analysis of lactic acid bacteria species in Miang, a post-fermented tea in Thailand, and their potential use as probiotics

**DOI:** 10.3389/fmicb.2024.1450158

**Published:** 2024-10-25

**Authors:** Masanori Horie, Supatjaree Ruengsomwong, Yoshihiro Ohmiya

**Affiliations:** ^1^Health and Medical Research Institute, National Institute of Advanced Industrial Science and Technology (AIST), Takamatsu, Kagawa, Japan; ^2^Expert Center of Innovative Herbal Products, Thailand Institute of Scientific and Technological Research (TISTR), Pathum Thani, Thailand; ^3^Biomedical Research Institute, AIST, Ikeda, Osaka, Japan

**Keywords:** Miang, lactic acid bacteria, *Lactiplantibacillus*, probiotics, diversity

## Abstract

**Introduction:**

Miang is one of the post-fermented teas made in Northern Thailand. Although lactic acid bacteria are involved in fermentation of Miang, details are still not clear. This study investigated the diversity of *Lactobacillaceae* bacteria, related to fermentation of Miang. Probiotic potential of isolated *Lactobacillaceae* bacteria was examined.

**Methods:**

*Lactobacillaceae* bacteria were isolated from 52 Miang samples collected from three provinces in northern Thailand and identified by MALDI-TOF MS. Hemolytic activity, antibiotic susceptibility, antimicrobial activity and tolerance to gastrointestinal juice were examined for probiotic potential of isolates.

**Results:**

A total of 1,181 *Lactobacillaceae* bacteria strains were isolated from Miang. The most abundant isolates were *Lactiplantibacillus pentosus*. Besides *Lactiplantibacillus plantarum*, *Levilactobacillus brevis*, *Paucilactobacillus suebicus*, *Lacticaseibacillus pantheris*, and *Secundilactobacillus collinoides* were also found with frequency. Of these isolates, 450 with a high score for MALDI-TOF identification were then screened for probiotic ability. Most isolates were resistant to aminoglycosides and clindamycin. Then, 35 isolates were tested for their antimicrobial activity against pathogens using the well diffusion method, and 31 isolates exhibited inhibition zones against *Staphylococcus aureus*, *Staphylococcus epidermidis*, *Salmonella enterica* serovar Typhimurium, *S. enterica* serovar Enteritidis, *Listeria monocytogenes*, *Propionibacterium acnes*, and *Streptococcus mutans*. All 31 isolates were non-hemolytic and readily tolerated simulated gastric juice at pH 3 and simulated intestinal juice at pH 8.

**Discussion:**

Miang contains lactic acid bacteria that could potentially be used as probiotics.

## 1 Introduction

Miang is a post-fermented tea produced in northern Thailand, especially in the provinces of Chiang Mai, Phrae, Chiangrai, Lampang, and Nan (Khanongnuch et al., 2017). The basic method of making Miang is as follows: (1) young soft tea leaves of *Camellia sinensis* var. *assamica* are picked; (2) the leaves are bundled and steamed; and (3) the steamed leaves are stuffed tightly into plastic bags and closed to create anaerobic conditions for lactate fermentation. The details of the Miang fermentation process differ between different regions of Thailand (Horie et al., 2020). There are two types of Miang depending on the fermentation period. One is “Miang-Faat (astringent Miang),” which has a shorter fermentation period (7–28 days) and a bitter taste, and the other is “Miang-Som (sour Miang),” which has a longer fermentation period (3–12 months) and a sour taste. Lactate fermentation is the main step in the production process of all types of Miang, and thus lactic acid bacteria (LAB) are essential for Miang production. Miang has beneficial physiological effects, and has been reported to exert antioxidant and anti-inflammatory properties (Abdullahi et al., 2021).

Post-fermented teas are produced in three areas of the world, namely border areas between Southeast Asian countries (Thailand, Laos and Myanmar), China [Yunnan (Puer tea), Hunan (Anhua dark tea), Hubei (Qingzhuan tea), Guangxi (Liubao tea), etc.] and Japan. One of the most well-known and widely produced post-fermented teas is Puer tea, primarily produced in Yunnan, China. Puer tea falls under the category of “dark tea” in China, a category which also includes teas produced in Sichuan and Hubei, China. Yunnan is the primary production area for puer tea (Guo et al., 2004). Puer tea has been reported to improve BMI and lipid profile and to exhibit prebiotic effects (Jensen et al., 2016; Liu et al., 2020). However, lactic acid fermentation does not include in the production process of puer tea. Lactic acid bacteria are not the primary fermenting bacteria in Chinese dark tea. Instead, puer tea undergoes aerobic fermentation, with filamentous fungi of the genus *Aspergillus* and yeasts being the major microorganisms involved in the fermentation process (Abe et al., 2008). Additionally, it has been reported that *Aspergillus niger* and *Blastobotrys adeninivorans* are involved in fermentation process of puer tea (Lyu et al., 2013). Similarly, Batabata-cha, one of the Japanese post-fermented tea is produced through aerobic fermentation involving *Aspergillus fumigatus*, and does not include lactic acid fermentation (Horie et al., 2019b). Tea extracts, including those from puer tea, have been shown to inhibit aflatoxin production by downregulating the transcription of aflatoxin synthesis-related genes *aflR* and *aflS* in *Aspergillus flavus* (Mo et al., 2013). Oxidized tea polyphenols also form complexes with aflatoxin B1, thereby inhibiting its absorption in the digestive tract (Lu et al., 2017). In contrast, Miang tea produced in Thailand, as well as Ishizuchi-kurocha and Awa-bancha tea produced in Japan, involve lactic acid fermentation in their production processes (Horie et al., 2020). The beneficial activities discussed above for Miang, have also been reported for Japanese post-fermented teas. Among Japanese post-fermented teas, the fermentation method for Awa-bancha, which is produced in Tokushima on Japan’s Shikoku island, is similar to that of Miang. Awa-bancha is produced only by lactate fermentation, mainly by *Camellia sinensis* var. *sinensis* (Nishioka et al., 2021). Japanese post-fermented teas, including Awa-bancha, have antioxidative activity (Horie et al., 2016). Furthermore, *Lactiplantibacillus plantarum* isolated from Awa-bancha exhibited an allergy-suppressing effect in a mouse model of atopic dermatitis (Yoshida et al., 2010). Another Japanese post-fermented tea, Ishizuchi-kurocha, which is produced by step-fermentation, had an inhibitory effect of degranulation of RBL-2H3 cells and lipid accumulation in adipocyte-like 3T3-L1 cells (Horie et al., 2019b). Additionally, Ishizuchi-kurocha contains gamma-amino butyric acid (GABA) and d-amino acids such as d-alanine, glutamate, and d-aspartate (Horie et al., 2023). These molecules are produced by LAB during fermentation. In many cases, the beneficial properties of post-fermented teas are derived from microorganisms related to fermentation. The role of LAB is particularly important. *Levilactobacillus brevis* strains isolated from Ishizuchi-kurocha and Awa-bancha have high GABA-producing ability (Horie et al., 2019b; Nishioka et al., 2022). In addition, *Lactiplantibacillus plantarum* and *Levilactobacillus brevis* produce D-amino acids. Furthermore, *Lactiplantibacillus plantarum* isolated from Ishizuchi-kurocha exhibits antibiotic resistance (Horie et al., 2019a), and *Limosilactobacillus fermentum* isolated from Miang exhibited antibacterial activity against pathogens such as *Listeria monocytogenes*, *Salmonella enterica* serovar Typhi, *Shigella sonnei*, and *Staphylococcus aureus*, in addition to an ability to scavenge free radicals (Klayraung and Okonogi, 2009). Thus, in post-fermented tea, the LAB involved in fermentation are affected by the environment during fermentation, producing many physiologically active substances that impart functionality to the post-fermented tea.

Therefore, LAB derived from post-fermented teas may have beneficial properties for probiotic use, and these LAB strains are expected to be useful in the food industry. However, the metabolism of physiologically active substances produced by LAB is species dependent. For example, *Levilactobacillus brevis* derived from Ishizuchi-kurocha exhibits high GABA-producing ability, whereas *Lactiplantibacillus plantarum* has low GABA-producing ability (Horie et al., 2019b). Additionally, even within the same species, different strains have different characteristics.

There have been several reports on the LAB species involved in the fermentation of Miang. The *Lactobacillus* species, *Lacticaseibacillus pantheris*, *Lactiplantibacillus pentosus*, *Paucilactobacillus suebicus*, and *Limosilactobacillus fermentum*, were reported from Miang obtained from markets in Bangkok and Chiang Mai (the production area was unknown), along with the novel species *Lacticaseibacillus thailandensis* and *Lacticaseibacillus camelliae*; however, *Lactiplantibacillus plantarum*, which is the dominant species in Japanese fermented teas, was not reported in these cases (Tanasupawat et al., 2007). According to another study, *Lactiplantibacillus pentosus* and *Lactiplantibacillus plantarum* were isolated from Miang produced in Chiang Mai, Chiang Rai, Nan, and Phrae, and the proportion of *Lactiplantibacillus pentosus* was reported to be high (Unban et al., 2021). These strains exhibited antibacterial activity, antibiotic resistance, and tolerance to gastric and intestinal juices. Additionally, *Lactiplantibacillus pentosus* derived from Miang has been reported to alleviate dextran sulfate sodium (DSS)-induced colitis (Kangwan et al., 2022). In another study, the dominant bacterial species in Miang, produced in eight provinces in upper-northern Thailand, were reported to be *Lactiplantibacillus plantarum* and *Lactiplantibacillus pentosus*. Of these two species, *Lactiplantibacillus pentosus* possessed a tannase gene and exhibited tannin tolerance, whereas *L. plantarum* did not possess a tannase gene and exhibited lower tannin tolerance (Chaikaew et al., 2017). Taken together, these results suggest that *Lactiplantibacillus pentosus* is an important species in Miang.

Miang is produced across a wide area of northern Thailand, and there is thought to be regional diversity. Among Japanese post-fermented teas, Ishizuchi-kurocha made in Ehime prefecture and Awa-bancha made in Tokushima prefecture differ in terms of the dominant LAB species (Nishioka et al., 2021; Horie et al., 2019a). The dominant species reported in Ishizuchi-kurocha is *Lactiplantibacillus plantarum*, and the dominant species in Awa-bancha is generally *Lactiplantibacillus pentosus*. Interestingly, the dominant species also differed between Awa-bancha that was produced in southern Tokushima (mainly *L. pentosus*) and eastern Tokushima (mainly *L. plantarum*) (Nishioka et al., 2021). It is not clear why the dominant species differ in the same post-fermented teas. The geographical area in which Miang is made is greater than Tokushima prefecture of Japan, and the geographical conditions, such as the climate, are different. The species of LAB involved in the fermentation of Miang differ depending on the production region, and it is thought that Miang-derived LAB may have potential as probiotics. If so, it may be possible to add some of the beneficial functions of Miang to other foods and supplements, with resulting health benefits.

In the present study, the LAB species involved in the fermentation of Miang produced in multiple regions of northern Thailand were investigated. The isolated *Lactobacillus* strains were then evaluated for their probiotic properties.

## 2 Materials and methods

### 2.1 Collection of samples

In total, 20 astringent-Miang and 32 sour-Miang samples from each step of the Miang production process including fresh leaves, steamed leaves, and fermented leaves at different fermentation times were collected from each sampling site. Except fresh leaves, each sample was thoroughly mixed before sampling. In case of fresh leaves, the leaves collected from miang trees grown in the same area were collected and pooled as one sample. Additionally, commercially supplied Miang samples available in wet markets were also collected. Fresh leaves were packed in zip lock plastic bags and kept in a foam box containing ice. In other cases, samples were aseptically added into a vial containing Viande–Levure (VL) broth (one sample/vial) and labeled as MSL with the sample number. For liquid samples, 5 mL of fermented liquid was mixed with 5 mL of double strength VL broth and labeled as MS with the sample number. All vials were tightly closed. After inoculation, the remaining samples were individually packed in plastic bags and kept in tightly closed containers as reference samples.

### 2.2 Microorganisms and growth conditions

The pathogenic indicator strains used in this study were *Staphylococcus aureus* ATCC 6538, *Staphylococcus epidermidis* TISTR 518, *Escherichia coli* ATCC 8379, *Salmonella enterica* serovar Typhimurium TISTR 292, *Salmonella enterica* serovar Enteritidis DMST 15676, *Listeria monocytogenes* DMST 13820, *Streptococcus mutans* ATCC 25175, *Propionibacterium acnes* DMST 14916, and *Candida albicans* ATCC 10231. All of the indicator microorganisms were stored at −80°C in 40% glycerol until use. *Staphylococcus aureus* ATCC 6538, *Staphylococcus epidermidis* TISTR 518, *E. coli* ATCC 8379, *Salmonella Typhimurium* TISTR 292, *Salmonella Enteritidis* DMST 15676, and *Listeria monocytogenes* DMST 13820 were grown in Mueller–Hinton (MH) agar or broth at 37°C for 18–24 h. *Streptococcus mutans* ATCC 25175 and *Propionibacterium acnes* DMST 14916 were anaerobically cultured on brain–heart infusion (BHI) agar or broth or 5% sheep blood agar at 37°C for 24–54 h. *Candida albicans* ATCC 10231 was grown in YMA (Merck, Darmstadt, Germany).

### 2.3 16S rRNA gene and ITS2 amplicon sequence analysis

Genomic DNA for microbiome analysis from Miang was prepared using the DNeasy® PowerLyzer^®^ PowerSoil^®^ kit (Qiagen GmbH, Hilden, Germany) in accordance with the manufacturer’s protocol with a bead cell disrupter (FastPrep-24™ Classic, MP Biomedicals, Irvine, CA, United States). Microbiome analysis was performed by Genome-Lead Corp. (Takamatsu, Japan). To analyze bacterial and fungal flora by amplicon sequence analysis, the V3–V4 region of the 16S rRNA gene and the internal transcribed spacer 2 (ITS2) region was amplified by KAPA HiFi HotStart (F. Hoffmann–La Roche, Ltd., Basel, Switzerland) using amplification primers 341F-806R and gITS7-ITS4 (Ihrmark et al., 2012), respectively. A library was created based on the 16S Metagenomics Sequencing Library Preparation kit (Illumina, Inc., San Diego, CA, United States), and the sequences were determined using Miseq (Illumina). The analysis was performed using QIIME 1.9.1.

### 2.4 Isolation and identification of LAB from samples

Although accuracy of bacterial identification at species- or strain levels requires additional genotypic methods, e.g., Species-specific primers PCR or Genome-based identification for calculation of % Average Nucleotide Identity (ANI), MALDI TOF was used for identification in this study without additional methods due to the following reasons: Huge number of purified isolates which were obtained from the samples. Only isolates with high confidence score values (>2.3) were selected for further study. Based on the Biotyper database, this level of score values are considered “highly reliable species identification. And MALDI TOF has been used for identification of Lactic acid bacteria in several publications (Angelakis et al., 2011; Dušková et al., 2012; Nacef et al., 2017; Kanak and Yolmaz, 2019; Kačániová et al., 2020; Bouchibane et al., 2022). All vials containing samples were incubated anaerobically in an anaerobic jar (Thermo Fisher Scientific™, United States) containing an AnaeroPack^®^ (Mitsubishi Gas Chemical Company, Inc., Tokyo, Japan) at 37°C for 24 h. After incubation, each sample was 10-fold serially diluted with normal saline. Then, 100 μL of each dilution was spread onto de Man, Rogosa, and Sharpe (MRS) agar (Merck), pH 6.8, supplemented with 0.05% L-cysteine HCl (Merck), and incubated anaerobically, as previously mentioned. For the master plates, 30 colonies/sample were randomly selected and streaked onto MRS agar plates containing 0.05% (w/v) L-cysteine. After incubation at 37°C for 24 h, each colony was further purified by cross-streaking and maintained at −80°C in 40% glycerol. Identification of isolated bacteria was performed by Matrix Assisted Laser Desorption/Ionization-Time of Flight Mass Spectrometry (MALDI-TOF MS). Sample preparation for MALDI TOF MS was carried out according to the “ethanol formic acid extraction” protocol (Bruker Daltonics, Germany). Three spots were prepared for each isolate. The target plate was directly analyzed using the Autoflex speed mass spectrometer with MALDI-Biotyper Realtime Classification version 4 (Bruker Daltonics) and MBT MSP Library version 6. The parameter settings were optimized for a mass range between 2 and 20 kDa. Spectra were recorded in the positive linear mode. A total of 200–500 laser shots were accumulated from each spot. Calibration of the mass spectrometer was performed with the Bruker’s bacterial test standard (*E. coli* DH5α extracts with the additional proteins RNase A and myoglobin, Bruker Daltonics). The results of analysis were expressed as confidence score values as follows: 2.300–3.000, highly reliable species identification; 2.000–2.299, highly reliable genus identification; 1.700–1.999, probable genus identification; and 0.000–1.699, no reliable identification.

### 2.5 Hemolytic assay

Each isolated LAB was streaked onto blood base agar supplemented with 5% sheep blood. After 24 h anaerobic incubation at 37°C, zones around the colonies were visualized and recorded. Only isolates showing no clear zones around colonies (*γ*-hemolysis) were considered non-hemolytic (Cafiso et al., 2012). *Staphylococcus aureus* ATCC 6538 was used as the positive control.

### 2.6 Antibiotic resistance phenotypes

Based on (EFSA Panel on Additives and Products or Substances used in Animal Feed (FEEDAP), 2018), nine types of standard antibiotic disks (Oxoid Limited, Basingstoke, United Kingdom) were used for the assay, namely ampicillin (10 μg), vancomycin (30 μg), chloramphenicol (30 μg), erythromycin (15 μg), tetracycline (30 μg), gentamicin (10 μg), kanamycin (10 μg), streptomycin (10 μg), and clindamycin (2 μg). Antibiotic susceptibility was determined using a disk diffusion assay according to the EUCAST disk diffusion test Vol 10.0, 2022. Briefly, the inoculum was prepared using the direct colony suspension method. Using a sterile cotton swab, 4–5 colonies of 18–24 h culture, grown anaerobically on MRS agar containing 0.05% L–cysteine HCl (w/v) at 37°C, were suspended in normal saline solution. After mixing, the suspension was further diluted with normal saline solution to obtain a turbidity match to that of the McFarland standard solution No. 0.5. To prepare test plates, the inoculum was thoroughly applied over the surface of MRS agar plates. Each standard antibiotic disk was then gently placed over the surface of the test plates (1 disk/plate). All plates were incubated anaerobically at 37°C for 24 h. After incubation, the inhibition zones for each antibiotic were measured and compared with the breakpoint values (Charteris et al., 1998a). The results were expressed as sensitive (S) or resistant (R). The test was performed in duplicate.

### 2.7 Antimicrobial activities of cell-free supernatants

As a cell-free supernatant, a 48 h culture of each isolate, grown in MRS broth, pH 6.8, supplemented with 0.05% L-*cysteine* HCl (w/v), was centrifuged at 10,000 rpm for 10 min at 4°C. The supernatant was collected, sterilized through a 0.22 μm filter membrane (Merck), then stored at −20°C until use. A well diffusion assay was used to determine the antimicrobial activities against pathogenic indicator microorganisms (Kazemipoor et al., 2012). Briefly, colonies of the pathogenic indicator microorganisms grown on suitable media were suspended in BHI broth (Merck). To prepare the inoculum, the turbidity of the microbial suspension was adjusted with normal saline solution to match that of a 0.5 McFarland standard. The inoculum was evenly applied onto the surface of 20 mL of either MHA, 5% sheep blood agar, or YM agar. Then, 6 mm wells were made in the test agar using a sterile cork borer. Each well was loaded with 60 μL of the cell-free supernatant. Normal saline was used as the negative control. After a suitable incubation period, the diameters of the inhibition zones observed around the wells were measured in millimeters. According to Shokryazdan et al. (2014), the effectiveness of antimicrobial activity was classified as follows: inhibition zones of more than 20 mm, 10–20 mm, and less than 10 mm were considered as strong, intermediate, and weak inhibition, respectively. The test was performed twice.

### 2.8 *In vitro* tolerance to simulated gastrointestinal juices

The abilities of LAB isolates to tolerate simulated gastric juice and simulated intestinal juice were assayed as reported previously by Charteris et al. (1998b). Briefly, LAB were cultured in MRS broth containing 0.05% L-cysteine HCl (w/v) and anaerobically incubated at 37°C for 18–24 h. After incubation, the cell pellet was harvested by centrifugation at 5,000 rpm for 10 min and subsequently washed twice with Dulbecco’s PBS. To prepare the inoculum, the washed cell pellet was suspended in Dulbecco’s PBS to obtain a cell concentration of approximately 10^9^ CFU/mL. Tolerance to simulated gastric juice: a volume of 100 μL of the inoculum was added into 5 mL of simulated gastric juice, pH 2 (6.2 g/L NaCl, 2.2 g/L KCl, 0.22 g/L CaCl_2_, 1.2 g/L NaHCO_3_, and 0.3% pepsin, w/v), which was then adjusted to pH 2 or pH 3. After mixing, the inoculated solution was incubated at 37°C for 90 min. Tolerance to simulated intestinal juice: A volume of 100 μL of the inoculum was added into 5 mL of simulated intestinal juice, pH 8 (0.03% Oxgall, 6.4 g/L NaHCO_3_, 0.239 g/L KCl, 128 g/L NaCl, and 0.1% pancreatin, w/v). After mixing, the inoculated solution was incubated at 37°C for 180 min. The number of LAB exposed to simulated gastrointestinal juices was enumerated at 0 and 90 min for simulated gastric juice and 90 and 180 min for simulated intestinal juice. Enumeration was performed by the pour plate method using MRS agar containing 0.05% L-cysteine HCl (w/v) as the growth medium. Following the incubation, the colonies in each plate were counted and calculated as CFU/mL. The survival rate was calculated using the following equation:


$$\%\mathrm{survival}=\frac{B\;X\;100\;}{A}$$

A = average number of bacteria detected at 0 min.

B = average number of bacteria detected at 90 or 180 min.

## 3 Results

### 3.1 Microbiome analysis of Miang

**Figure 1:**
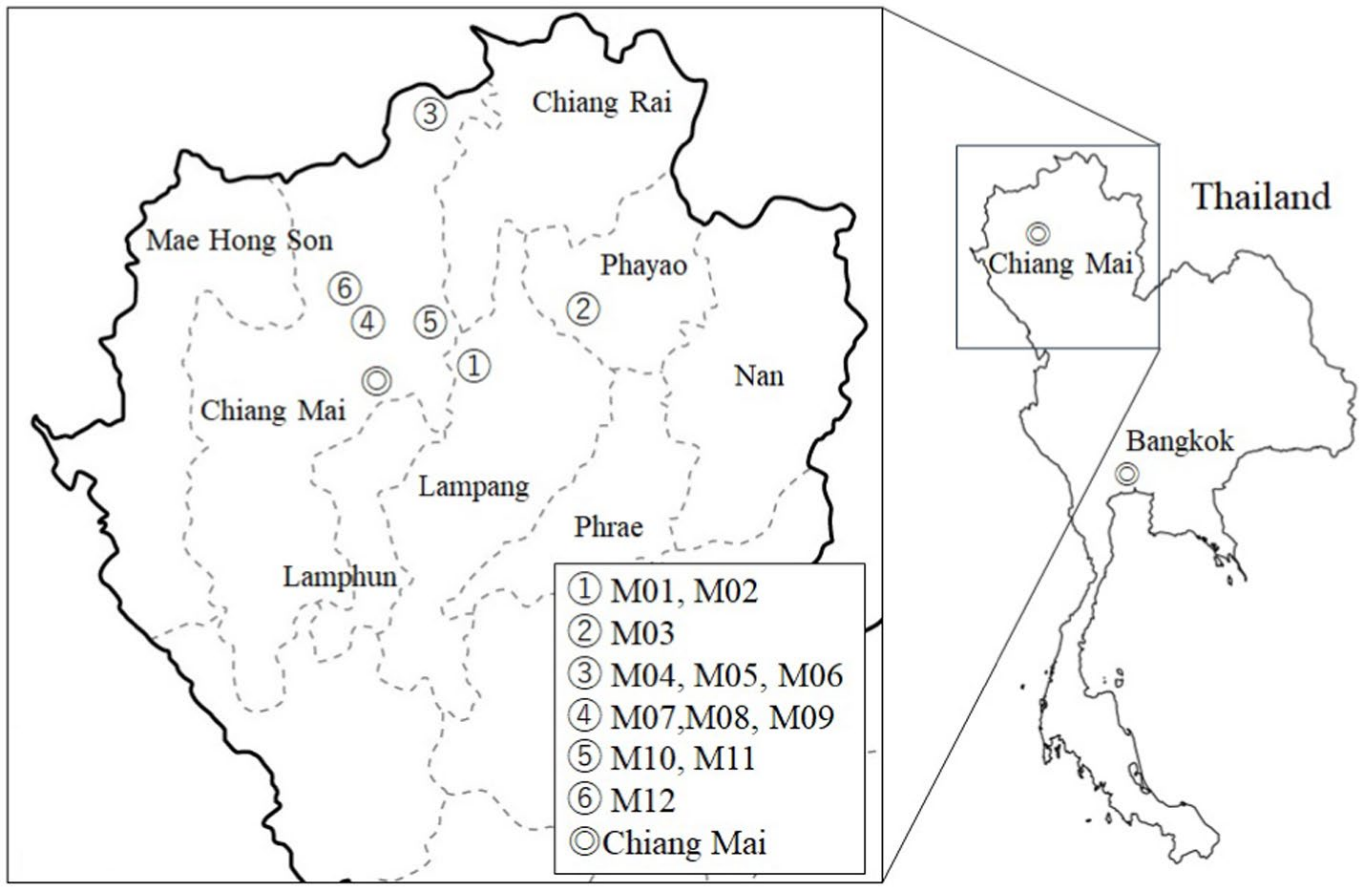
Location of Miang sampling point for microbiome analysis.

**Figure 2:**
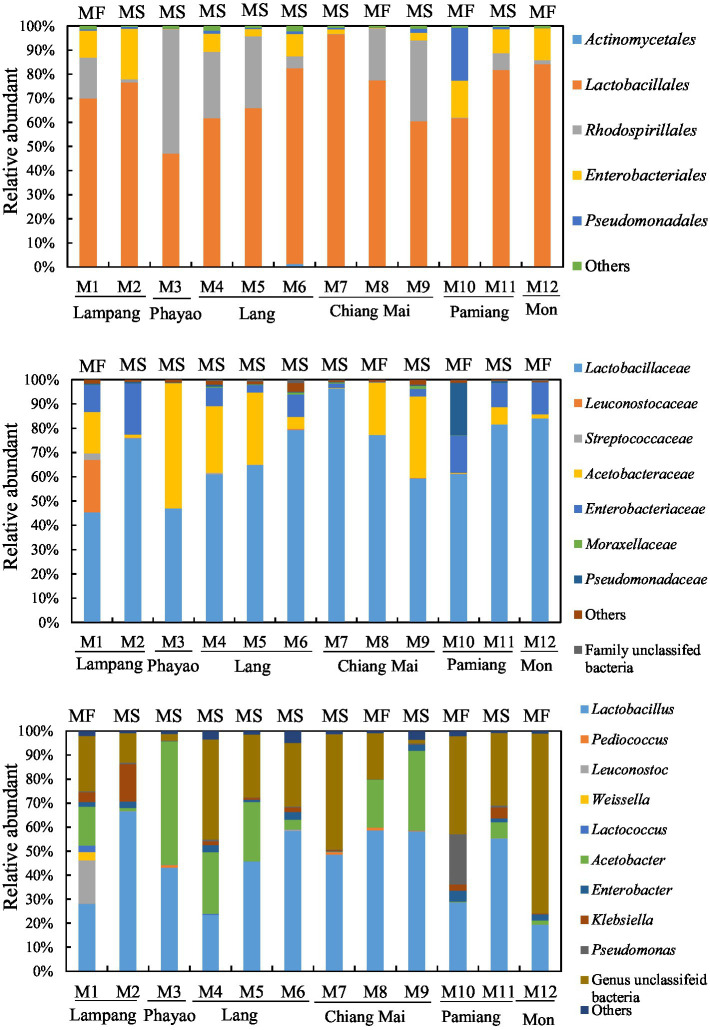
Bacterial flora of Miang below the order level. Upper, middle, and lower graphs show the results of bacterial flora at the order, family, and genus levels, respectively. DNA was extracted from Miang samples and the bacterial flora was analyzed by 16S rRNA gene amplicon sequencing. “Others” includes orders, families, or genera that accounted for less than 1% in all samples. “MF” and “MS” indicate “Miang-Faat” and “Mian-Som,” respectively.

**Figure 3:**
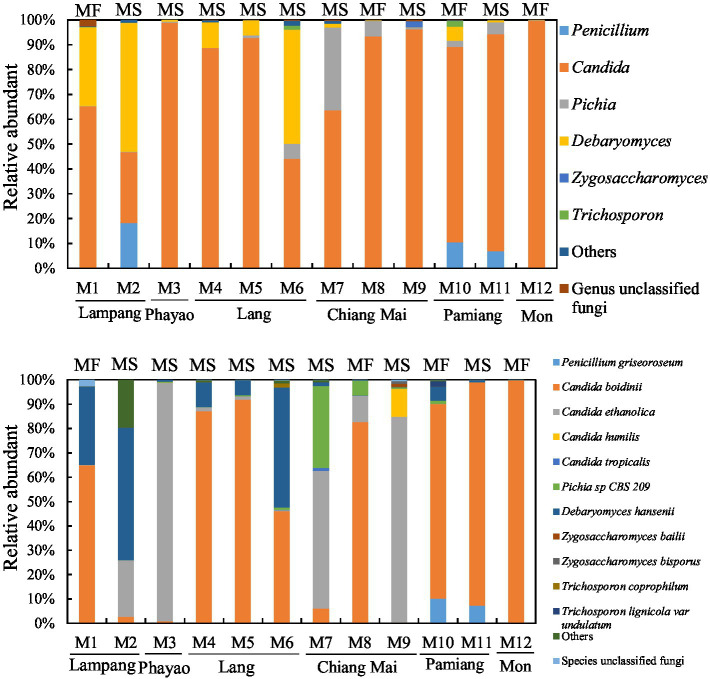
Fungal flora of Miang below the family level. Upper and lower graphs show the results of fungal flora at the genus and species level, respectively. DNA was extracted from the Miang samples, and the fungal flora was analyzed by ITS2 amplicon sequencing. “Others” includes orders, families, or genera that accounted for less than 1% in all samples. “MF” and “MS” indicate “Miang-Faat” and “Mian-Som,” respectively.

The 16S rRNA gene and ITS2 amplicon sequence information for the Miang samples is shown in Table 1 and Figure 1. According to the 16S rRNA gene amplicon sequence, *Lactobacillales* was dominant at the order level, except for one sample obtained from Phayao. The proportion of *Lactobacillales* was approximately 60–80% (Figure 2). One sample from Chiang Mai had the highest percentage of *Lactobacillales* (96.5%). The lowest percentage of *Lactobacillales*, which was found in the Phayao sample, was 47%. *Rhodospirillales* was the second most dominant after *Lactobacillales* at the order level. In one Miang sample obtained from Phayao, *Rhodospirillales* predominated at 51.6%. At the genus level, the Miang from Phayao contained 43% *Lactobacillus* and 51.5% *Acetobacter*. As this ratio was almost the same as that of *Lactobacillales* and *Rhodospirillales*, it was considered that the *Lactobacillales* comprised *Lactobacillus* and the *Rhodospirillales* comprised *Acetobacter*. Among the identifiable genera, the genus *Lactobacillus* was detected most frequently, followed by *Acetobacter*. The ratio of *Lactobacillus* was often approximately 40–60%. In recent years, the genus *Lactobacillus* has been reclassified into 25 new genera based on its genome (Zheng et al., 2020), but at the time of this study, the new classification of genera was unknown. In contrast to the genus *Lactobacillus*, the proportions of *Lactococcus* and *Leuconostoc* were low, at approximately 0–0.5% and 0–0.4%, respectively. *Lactococcus*, *Leuconostoc*, and *Weissella* accounted for 2.7, 18, and 3.5%, respectively, in only one Miang sample obtained in Lampag. The proportion of *Lactobacillus* in Miang was low, at 28.1%. *Weissella* was rarely found in the Miang samples. According to the ITS region amplicon sequence, *Candida* was the dominant species among the Miang fungal flora in 13 of 15 samples (Figure 3). In two samples, *Debaryomyces* was the dominant species. The percentage of *Candida* exceeded 90% in five samples. In Miang containing the highest percentage of *Candida* species, the percentage was 99.6%. The proportions of *Debaryomyces* in Miang were high (31.7, 45.9, and 51.9%). The dominant species of *Candida* differed depending on the isolation area. The dominant fungal species in Miang obtained from Phayao and Chiang Mai was *Candida ethanolica*, whereas in all other samples the dominant species was *Candida boidinii*. The proportion of *Pichia* among Miang samples was 33.3%. In addition, approximately 7–18% of *Penicillium* was found in three samples. In the present study, no difference was found in the microflora of Miang according to production region. In addition, there was no difference in flora between Miang-Faat and Miang-Som.

**Table 1:** Details of Miang for 16S rRNA gene and ITS2 amplicon sequence.

Code in this study	Type of sample	Description of sample	pH	Sampling region	Sampling sites
M01	Astringent miang	Steamed leaves in water and pickling salt, anaerobically fermented 2–3 days	4	Lampang	Mountainous plantation area in Ban Pa Miang, Mueang Pan district, Lampang Geographic coordinates: 18°49′53.1”N 99°23′15.5″E
M02	Sour miang	Steamed leaves in water and pickling salt, anaerobically fermented >15 days	4		
M03	Sour miang	fermented for >1 month from vendor No. 4	4	Phayao	“Mae Thong Khum” wet market, Phayao Geographic coordinates: 19°08′21.9”N 99°54′41.4″E
M04	Sour miang	Sour miang fermented for 2 days	4	Lang	House of local miang producer No. 4 on Doi Lang, Mae Ai district, Chiang Mai Geographic coordinates: 20°04′55.1”N 99°17′40.2″E
M05	Sour miang	Sour miang fermented for 15 days	4		
M06	Sour miang	Sour miang fermented for 20 days	4		
M07	Sour miang	Sour miang from vendor No. 4	4	Chiang Mai	Mae Malai wet market, Mae Rim district, Chiang Mai Geographic coordinates: 19°05′41.3”N 98°56′09.9″E
M08	Astringent miang	Astringent miang from vendor No. 5	4		
M09	Sour miang	Sour miang from vendor No. 6	4		The wet market opposite to 7th Artillery Battalion Prapinklao camp, Mae Rim district, Chiang Mai
M10	Astringent miang	Astringent miang, fermented for 2 days	4	Pamiang	House of local miang producer No. 5, community supported by “Pa-Miang” Royal Project Development Center Doi Saket district, Chiang Mai
M11	Sour miang	Sour miang, fermented for 10 days	4		
M12	Astringent miang	Astringent miang, fermented for 4 days	n.t.	Mon Ngo	House of local miang producer No. 6, Banlao Patana community supported by “Mon Ngo” Royal Project Development Center Mae Tang district, Chiang Mai Geographic coordinates: 19°10′37.2”N 98°47′50.5″E

### 3.2 Isolation and identification of LAB from Miang samples

Fifty-two samples of Miang were used for LAB isolation using the dilution method and the spread plate technique on MRS agar supplemented with 0.03% (w/v) L-cysteine HCl. In total 1,181 purified isolates were obtained. All isolates were catalase-negative, Gram-positive rods. The results of identification by MALDI-TOF MS revealed that *Lactiplantibacillus* spp. accounted for the majority of the strains (Table 2). Out of 1,181 isolates, 889 isolates (75.28%) were identified as *L. pentosus*; 136 isolates (11.52%) as *L. plantarum*; 59 isolates (4.99%) as *Levilactobacillus brevis*; 39 isolates (3.30%) as *Paucilactobacillus suebicus*; 25 isolates (2.12%) as *Lacticaseibacillus pantheris*; 21 isolates (1.78%) as *Secundilactobacillus collinoides*; 4 isolates (0.34%) as *Levilactobacillus acidifarinae*; 3 isolates (0.25%) as *Lacticaseibacillus paracasei;* 3 isolates (0.25%) as *Paucilactobacillus vaccinostercus*, and 2 isolates (0.17%) as *Lentilactobacillus parabuchneri.* The score values of the identification ranged from 1.7–3–2. To obtain isolates with a high confidence level for species identification, only 450 isolates with a score ≥ 2.3 were selected for subsequent characterization of probiotic properties.

**Table 2:** Identification of isolated lactic acid bacteria by culture method.

	Sampling region of Miang							
	Lampang	Phayao	Lang	Chiang Mai	Pamiang	Mon	Nan	Total number
*Lactiplantibacillus pentosus*	51	164	151	81	94	48	300	889
*Lactiplantibacillus plantarum*	18	11	5	8	30	2	62	136
*Levilactobacillus brevis*	17	18	n.d.	14	n.d.	1	9	59
*Paucilactobacillus suebicus*	2	5	6	15	17	n.d.	4	39
*Lacticaseibacillus pantheris*	10	n.d.	2	1	4	n.d.	8	25
*Secundilactobacillus collinoides*	n.d.	n.d.	n.d.	19	n.d.	n.d.	2	21
*Levilactobacillus acidifarinae*	n.d.	1	n.d.	n.d.	n.d.	n.d.	3	4
*Lacticaseibacillus paracasei*	2	n.d.	n.d.	n.d.	n.d.	n.d.	1	3
*Paucilactobacillus vaccinostercus*	n.d.	n.d.	n.d.	1	n.d.	1	1	3
*Lentilactobacillus parabuchneri*	n.d.	2	n.d.	n.d.	n.d.	n.d.	n.d.	2

### 3.3 Hemolytic assay

Evaluation of the safety of potential probiotics was first performed by a hemolysis assay. Of the 1,181 LAB isolates, 994 isolates (84.2%) exhibited hemolytic activity with clear zones around the colonies when grown on blood agar, while 187 isolates (15.8%) did not show hemolytic activity.

### 3.4 Antibiotic resistance phenotypes

Table 3 shows the antibiotic resistance profiles of the 450 LAB isolates. All isolates were sensitive to ampicillin and chloramphenicol, and resistant to vancomycin. Most of the LAB isolates were resistant to aminoglycosides and clindamycin. It was found that 437 isolates (97.11%), 448 isolates (99.56%), 447 isolates (99.35%), and 413 isolates (91.78%) were resistant to gentamycin, kanamycin, streptomycin, and clindamycin, respectively. Eight isolates (1.78%) were resistant to tetracycline, whereas 2 isolates (0.44%) were resistant to erythromycin. Previous reports have indicated that antibiotic resistance to aminoglycosides (gentamycin, kanamycin, and streptomycin) is intrinsic in LAB isolates (Elkins and Mullis, 2004; Toomey et al., 2010; Nawaz et al., 2011; Solieri et al., 2014), as is vancomycin resistance (Gueimonde et al., 2013; Fraqueza, 2015; Delcour et al., 1999). Since intrinsic resistance is not transferable, this type of resistance is not a safety concern. By contrast, resistance to erythromycin, tetracycline, and clindamycin is classified as acquired resistance able to transfer via mobile genetic elements (Charteris et al., 1998b; van Hoek et al., 2011; Campedelli et al., 2018). Hence, LAB isolates with acquired antibiotic resistance were excluded from further analysis. In this study, only 35 isolates susceptible to ampicillin, chloramphenicol, erythromycin, tetracycline, and clindamycin were selected for further study (Table 4).

**Table 3:** Antibiotic susceptibility of 450 LAB isolates.

Types of antibiotic	Number of resistant isolates to each antibiotic
Ampicillin	0
Tetracyclin	8
Vancomycin	450
Streptomycin	447
Chloramphenicol	0
Gentamicin	437
Kanamycin	448
Clindamycin	413
Erythromycin	2

**Table 4:** Antibiotic resistance profiles of LAB isolated from Miang samples.

			Antibiotics								
No.	Code	Bacteria	Ampicillin	Vancomycin	Chloramphenicol	Erythromycin	Tetracycline	Gentamicin	Kanamycin	Streptomycin	Clindamycin
1	MSL 1–24	*L. pentosus*	S	R	S	S	S	R	R	R	S
2	MSL 15–3	*L. brevis*	S	R	S	S	S	R	R	R	S
3	MSL 17–22	*L. pentosus*	S	R	S	S	S	R	R	R	S
4	MSL 21–15	*L. pentosus*	S	R	S	S	S	R	R	R	S
5	MS 1–12	*L. paracasei*	S	R	S	S	S	R	R	R	S
6	MS 2–8	*L. pentosus*	S	R	S	S	S	R	R	R	S
7	MS 2–22	*L. pentosus*	S	R	S	S	S	R	R	R	S
8	MS 3–14	*L. pentosus*	S	R	S	S	S	R	R	R	S
9	MS 4–7	*L. pentosus*	S	R	S	S	S	R	R	R	S
10	MS 4–18	*L. pentosus*	S	R	S	S	S	R	R	R	S
11	MS 4–24	*L. pentosus*	S	R	S	S	S	R	R	R	S
12	MS 5–4	*L. pentosus*	S	R	S	S	S	R	R	R	S
13	MS 5–14	*L. pentosus*	S	R	S	S	S	R	R	R	S
14	MS 6–5	*L. pentosus*	S	R	S	S	S	R	R	R	S
15	MS 10–1	*L. pentosus*	S	R	S	S	S	R	R	R	S
16	MS 10–8	*L. pentosus*	S	R	S	S	S	R	R	R	S
17	MS 10–24	*L. pentosus*	S	R	S	S	S	R	R	R	S
18	MS 11–1	*L. brevis*	S	R	S	S	S	R	R	R	S
19	MS 13–23	*L. plantarum*	S	R	S	S	S	R	R	R	S
20	MS 13–24	*L. pentosus*	S	R	S	S	S	R	R	R	S
21	MS 14–4	*L. brevis*	S	R	S	S	S	R	R	R	S
22	MS 16–5	*L. paracasei*	S	R	S	S	S	R	R	R	S
23	MS 16–6	*L. paracasei*	S	R	S	S	S	R	R	R	S
24	MS 16–13	*L. pentosus*	S	R	S	S	S	R	R	R	S
25	MS 19–11	*L. brevis*	S	R	S	S	S	S	R	R	S
26	HR 1–2	*L. pentosus*	S	R	S	S	S	R	R	R	S
27	HR 1–10	*L. pentosus*	S	R	S	S	S	S	R	R	S
28	HR 2–5	*L. pentosus*	S	R	S	S	S	R	R	R	S
29	HR 4–1	*L. pentosus*	S	R	S	S	S	R	R	R	S
30	HR 7–3	*L. pentosus*	S	R	S	S	S	R	R	R	S
31	HR 8–2	*L. pentosus*	S	R	S	S	S	R	R	R	S
32	HR 12–1	*L. pentosus*	S	R	S	S	S	R	R	R	S
33	HR 18–5	*L. pentosus*	S	R	S	S	S	R	R	R	S
34	HR 19–2	*L. pentosus*	S	R	S	S	S	R	R	R	S
35	HR 29–2	*L. pentosus*	S	R	S	S	S	R	R	R	S

S, sensitive; R, resistant.

### 3.5 Antimicrobial properties

The antimicrobial activities of 35 selected LAB isolates were assayed against nine pathogens using the well diffusion method. Most of the LAB isolates showed intermediate inhibitory activity (Tables 5, 6). No isolates could inhibit *Candida albicans* ATCC 10231. Twenty eight isolates (MSL 1–24, MSL 17–22, MSL 21–15, MS 1–12, MS 2–8, MS 2–22, MS 3–14, MS 4–7, MS 4–18, MS 4–24, MS 5–4, MS 6–5, MS 10–1, MS 10–8, MS 10–24, MS 13–23, MS 13–24, MS 16–5, MS 16–6, MS 16–13, MS 19–11, HR 1–2, HR 4–1, HR 7–3, HR 8–2, HR 12–1, HR 18–5, and HR 19–2) possessed inhibitory activity against *Staphylococcus aureus* ATCC 6538 (inhibition zone 11 to 26 mm), *Escherichia coli* ATCC 8379 (inhibition zone 11 to 20 mm), *Salmonella enterica* serovar Typhimurium TISTR 292 (inhibition zone 12 to 30 mm), *Salmonella enterica* serovar Enteritidis DMST 15676 (inhibition zone 12 to 23 mm), *Listeria monocytogenes* DMST 13802 (inhibition zone 11 to 20 mm), *Staphylococcus epidermidis* TISTR 518 (inhibition zone 11 to 22 mm), *Propionibacterium acnes* DMST 14961 (inhibition zone 11 to 18 mm), and *Streptococcus mutans* ATCC 25175 (inhibition zone 9 to 18 mm). MSL 15–3, HR 2–9, and HR 29–2 exhibited inhibitory activity against *Staphylococcus aureus* ATCC 6538, *Salmonella enterica* serovar Typhimurium TISTR 292, *Salmonella enterica* serovar Enteritidis DMST 15676, *Listeria monocytogenes* DMST 13802, *Staphylococcus epidermidis* TISTR 518, *Propionibacterium acnes* DMST 14961, and *Streptococcus mutans* ATCC 25175, but not against *Escherichia coli* ATCC 8379; while MS 14–4 showed antagonistic activity only against *Salmonella enterica* serovar Typhimurium TISTR 292. A comparison of the diameters of the inhibition zones revealed that most of the LAB isolates exhibited intermediate inhibitory activity (Table 6). This result was in agreement with the work of Klayraung and Okonogi (2009) who reported that *Limosilactobacillus fermentum* FTL10BR and *Limosilactobacillus fermentum* FTL2311 isolated from Miang had inhibitory activity toward *Staphylococcus aureus* DMST 6512 (inhibition zone 8 to 10 mm) and *Listeria monocytogenes* DMST 17303 (inhibition zone 10 mm).

**Table 5:** Number of LAB isolates showing different levels of antagonistic activity against 9 pathogens.

Tested pathogens	No. of LAB isolates showing different levels of inhibitory activity			
	High	Intermediate	Low	No
**Skin pathogens**				
*C. albicans* ATCC 10231	–	–	–	35
*P. acnes* DMST 14961	–	32	3	
*S. epidermidis* TISTR 518	3	29	–	3
**Oral pathogenic bacterium**				
*S. mutans* ATCC 25175	–	29	3	3
**Foodborne pathogenic bacteria**				
*S. aureus* ATCC 6538	12	20	–	3
*E. coli* ATCC 8379	–	29	–	6
*S. typhimurium* TISTR 292	10	23	–	2
*S. enteritidis* DMST 15676	5	27	–	3
*L. monocytogenes* DMST 13802	1	31	–	3

**Table 6:** Antimicrobial activity of LAB isolates from Miang samples.

No.	Code	Bacteria	Diameter of clear zone (mm)								
			*S. aureus*	*E. coli*	*S. typhimurium*	*S. enteritidis*	*L. monocytogenes*	*S. epidermidis*	*P. acnes*	*C. albicans*	*S. mutans*
1	MSL 1–24	*L. pentosus*	18	17	26	20	17	18	12	0	13
2	MSL 15–3	*L. brevis*	11	0	12	15	17	14	16	0	16
3	MSL 17–22	*L. pentosus*	20	18	28	18	20	19	14	0	13
4	MSL 21–15	*L. pentosus*	17	13	13	12	11	11	11	0	14
5	MS 1–12	*L. paracasei*	19	14	15	13	12	12	13	0	10
6	MS 2–8	*L. pentosus*	17	15	17	17	15	15	16	0	14
7	MS 2–22	*L. pentosus*	26	17	21	20	16	19	15	0	13
8	MS 3–14	*L. pentosus*	21	14	17	16	15	26	14	0	14
9	MS 4–7	*L. pentosus*	21	15	19	18	15	18	15	0	12
10	MS 4–18	*L. pentosus*	23	15	19	17	15	17	17	0	11
11	MS 4–24	*L. pentosus*	22	15	20	18	16	18	14	0	11
12	MS 5–4	*L. pentosus*	20	13	17	15	13	17	13	0	9
13	MS 5–14	*L. pentosus*	20	11	15	14	12	12	11	0	13
14	MS 6–5	*L. pentosus*	21	12	16	16	11	13	12	0	12
15	MS 10–1	*L. pentosus*	25	15	19	19	26	20	16	0	13
16	MS 10–8	*L. pentosus*	25	15	18	18	16	19	15	0	13
17	MS 10–24	*L. pentosus*	24	15	17	17	17	19	15	0	14
18	MS 11–1	*L. brevis*	0	0	0	0	0	0	0	0	0
19	MS 13–23	*L. plantarum*	19	12	15	16	13	16	18	0	13
20	MS 13–24	*L. pentosus*	25	19	30	22	20	22	14	0	15
21	MS 14–4	*L. brevis*	0	0	18	0	0	0	0	0	0
22	MS 16–5	*L. paracasei*	12	16	27	19	15	18	13	0	14
23	MS 16–6	*L. paracasei*	12	15	26	17	14	17	13	0	13
24	MS 16–13	*L. pentosus*	18	14	15	15	12	15	13	0	11
25	MS 19–11	*L. brevis*	29	16	21	20	17	19	13	0	10
26	HR 1–2	*L. pentosus*	20	17	28	19	18	18	13	0	14
27	HR 1–10	*L. pentosus*	0	0	0	0	0	0	0	0	0
28	HR 2–5	*L. pentosus*	20	0	27	19	18	19	14	0	12
29	HR 4–1	*L. pentosus*	18	20	20	21	19	19	18	0	17
30	HR 7–3	*L. pentosus*	12	12	18	20	14	15	17	0	17
31	HR 8–2	*L. pentosus*	12	16	13	23	18	19	12	0	17
32	HR 12–1	*L. pentosus*	18	17	15	23	20	21	18	0	18
33	HR 18–5	*L. pentosus*	15	11	12	11	14	16	12	0	16
34	HR 19–2	*L. pentosus*	16	13	16	15	15	18	16	0	17
35	HR 29–2	*L. pentosus*	22	0	29	21	20	20	15	0	12

*S. aureus ATCC 6538; E. coli ATCC 8379; S. typhimurium TISTR 292; S. enteritidis DMST 15676; L. monocytogenes DMST 13802; S. epidermidis TISTR 518; P. acnes DMST 14961; C. albicans ATCC 10231; S. mutans ATCC 25175.*

### 3.6 *In vitro* tolerance to gastric and intestinal juices

The selected LAB isolates were investigated for tolerance to simulated gastric juice at pH 2 and pH 3, and simulated intestinal juice containing 0.3% (w/v) of bile salt. The results are presented in Table 7.

**Table 7:** Tolerance of LAB isolated from Miang samples to simulated gastrointestinal juice.

No.	Code	Bacteria	% survival			
			Simulated gastric juice		Simulated intestinal juice	
			pH2	pH3	90 min	180 min
1	MSL 1–24	*L. pentosus*	0	71.5	98.8	102.2
2	MSL 15–3	*L. brevis*	0	94.2	100.0	96.6
3	MSL 17–22	*L. pentosus*	0	92.7	95.3	94.8
4	MSL 21–15	*L. pentosus*	0	62.9	84.8	92.7
5	MS 1–12	*L. paracasei*	0	100.5	105.6	105.1
6	MS 2–8	*L. pentosus*	0	0.0	89.3	101.4
7	MS 2–22	*L. pentosus*	0	84.1	96.9	95.3
8	MS 3–14	*L. pentosus*	0	52.3	99.9	99.6
9	MS 4–7	*L. pentosus*	0	50.9	98.7	98.7
10	MS 4–18	*L. pentosus*	0	97.8	97.5	98.0
11	MS 4–24	*L. pentosus*	0	96.4	91.2	83.1
12	MS 5–4	*L. pentosus*	0	97.9	99.5	98.1
13	MS 5–14	*L. pentosus*	0	86.5	93.8	93.8
14	MS 6–5	*L. pentosus*	0	90.4	94.9	94.4
15	MS 10–1	*L. pentosus*	0	100.4	102.4	103.8
16	MS 10–8	*L. pentosus*	0	98.9	100.0	99.6
17	MS 10–24	*L. pentosus*	0	99.7	97.0	97.0
18	MS 11–1	*L. brevis*	0	87.1	98.5	98.4
19	MS 13–23	*L. plantarum*	0	92.3	92.1	91.0
20	MS 13–24	*L. pentosus*	0	78.7	95.6	95.3
21	MS 14–4	*L. brevis*	0	92.9	99.3	99.9
22	MS 16–5	*L. paracasei*	0	93.5	91.4	93.9
23	MS 16–6	*L. paracasei*	0	94.8	98.3	94.8
24	MS 16–13	*L. pentosus*	0	81.0	66.4	69.1
25	MS 19–11	*L. brevis*	0	94.8	100.2	99.3
26	HR 1–2	*L. pentosus*	0	93.0	102.1	101.0
27	HR 1–10	*L. pentosus*	0	76.7	99.1	98.8
28	HR 2–5	*L. pentosus*	0	86.6	102.7	97.9
29	HR 4–1	*L. pentosus*	0	89.6	95.8	96.7
30	HR 7–3	*L. pentosus*	0	100.4	97.3	96.6
31	HR 8–2	*L. pentosus*	0	80.9	98.9	98.9
32	HR 12–1	*L. pentosus*	0	89.4	100.7	101.3
33	HR 18–5	*L. pentosus*	0	93.2	94.4	97.2
34	HR 19–2	*L. pentosus*	0	90.9	96.8	97.4
35	HR 29–2	*L. pentosus*	0	58.0	120.3	126.0

Simulated gastric juice = normal saline containing 0.3% pepsin. Simulated intestinal juice = normal saline containing 0.3% bile salt and 0.1% pancreatin.

It was found that none of the 35 LAB isolates could survive after 90 min exposure to simulated gastric juice at pH 2. A possible explanation for this phenomenon might be that this pH is harsher than the natural environmental niche from which these LAB were isolated. The pH values of the fermented liquid of Miang samples were between 3 and 4 implying that *Lactobacillus* in Miang might not survive in a pH lower than 3. When tested with simulated gastric juice at pH 3, the survival rates of the 34 isolates were higher than those at pH 2.0. Nineteen isolates (MSL 15–3, MSL 17–22, MS 1–12, MS 4–18, MS 4–24, MS 5–4, MS 6–5, MS 10–1, MS 10–8, MS 10–24, MS 13–23, MS 14–4, MS 16–5, MS 16–6, MS 19–11, HR 1–2, HR 7–3, HR 18–5, and HR 19–2) showed survival rates of ≥90%. MSL 1–12, MS 10–1, and HR 7–3 showed maximum tolerance, with 100% survival, whereas MS 2–8 was unable to survive.

Based on their ability to tolerate simulated intestinal juice at 90 and 180 min contact time, the selected LAB isolates could be categorized into three groups: (1) those with a higher survival rate at 180 min than 90 min (e.g., MSL 21–15 and MS 2–8); (2) those with a higher survival rate at 90 min than 180 min (e.g., MS 4–24); and (3) those with comparable survival rates at 90 min and 180 min (32 isolates). HR 29–2 exhibited the highest tolerance with 100% survival, whereas MS 16–13 only showed survival of 66%. Although LAB isolates were killed at pH 2, the survival of 34 LAB isolates at pH 3 in the presence of 0.3% bile salts for 3 h was sufficient to be regarded as probiotic candidates. It should be noted that MS 2–8 was excluded because it did not survive at pH 3.

## 4 Discussion

In this study, microbiome analysis was first performed on Miang obtained from seven different production areas in northern Thailand. No differences were found in the Miang microbiota between the production regions. Currently, while it is possible to identify microorganisms in fermented foods, distinguishing local products from similar items produced in other regions remains challenging. Despite minor regional variations in the production process of Miang, the key fermentation conditions—such as temperature, type of tea leaves, and anaerobic environment—are consistent across regions. These consistent fermentation conditions are likely to lead to similar microbial floras. Consequently, the similarity in fermentation conditions contributes to the similarity observed in the microbial flora. In most Miang, LAB of the genera *Lactobacillus* and *Acetobacter* were dominant. These two bacteria are important for the fermentation of Miang. A previous study reported that 39.7–79.5% of Miang flora, prepared in Papae district, Chiang Mai, belonged to the family *Lactobacillaceae*, and they concluded that *Lactobacillus* (29.2–77.2%) and *Acetobacter* (3.8–22.8%), and the unicellular fungi *Candida* (72.5–89.0%) and *Pichia* (8.1–14.9%) are important microorganisms for Miang fermentation (Unban et al., 2020). The study by Unban et al. (2020) was conducted separately at approximately the same time as the present study, and the results are largely consistent. All findings therefore indicate that *Lactobacillus* and *Acetobacter* are important bacteria and *Candida* is an important fungus in Miang fermentation. Additionally, according to the identification of isolated LAB, it was clear that *Lactiplantibacillus pentosus* is a dominant species among Miang samples. This finding was different from previous studies that reported *Lactiplantibacillus. plantarum* to be the dominant species in all Miang fermentations (Chaikaew et al., 2017; Okada et al., 1986). It should be noted that certain species (*Levilactobacillus brevis*, *Secundilactobacillus collinoides*, *Levilactobacillus acidifarinae*, *Lacticaseibacillus paracasei, Paucilactobacillus vaccinostercus*, and *Lentilactobacillus parabuchneri*) detected in Miang samples in the present study, were not observed in other studies (Tanasupawat et al., 2007; Okada et al., 1986). These inconsistent findings might be due to differences in sampling sites, the medium used for isolation, the criteria for colony selection, or the number of isolates or methods used for identification.

*Debaryomyces* and *Pichia* may also be necessary for Miang production. Interestingly, the microbial composition that appears to be important for the fermentation of Miang differs from that of Japanese post-fermented teas. The genus *Lactiplantibacillus* was dominant in Awa-bancha, and *Paucilactobacillus*, *Secundilactobacillus*, and *Ligilactobacillus* were also identified. Additionally, *Klebsiella* spp. are frequently found. In one case, the proportion of *Klebsiella* was close to 50%. However, the proportion of *Acetobacter* was low and its detection was rare (Nishioka et al., 2021). In Ishizuchi-kurocha, another Japanese post-fermented tea, *Lactobacillus* was reported to be dominant, and *Pseudomonas* and *Klebsiella* were often also present, whereas *Acetobacter* spp. were rarely found (Ohno et al., 2024). The dominant fungal flora of Ishizuchi-kurocha was *Aspergillus* spp. (Ohno et al., 2024; Mizuno et al., 2020); however, the fungal flora of Awa-bancha remains to be determined. The production method differs between Miang and Ishizuchi-kurocha. Ishizuchi-kurocha is produced by a two-step fermentation. The primary fermentation is aerobic fermentation mainly mediated by *Aspergillus* spp., followed by anaerobic fermentation mainly mediated by *Lactiplantibacillus*. During anaerobic fermentation of Ishizuchi-kurocha, *Pichia* spp. were detected (Mizuno et al., 2020; Yamamoto et al., 2019). *Aspergillus* spp. are important fungi in Japanese post-fermented teas. Three of the four types of traditional Japanese post-fermented teas are fermented by *Aspergillus*. Whereas, *Candida* spp. were not dominant in any of the Japanese post-fermented teas (Ohno et al., 2024; Mizuno et al., 2020). The fungal flora involved in the fermentation process differed substantially between the Japanese and Thai post-fermented teas. *Aspergillus flavus* is known to produce aflatoxins in tropical regions (Ehrlich et al., 2007). The invasion of *A. flavus* into fermented foods in the tropics poses a health risk. Therefore, in tropical regions, the use of *Aspergillus* spp. in food is risky. This has meant that while *Aspergillus* is widely used for fermentation in temperate zones, it has generally been avoided in the tropics. The LAB isolated from Miang showed some characteristic properties that may be advantageous for their application as probiotics. Some of the isolated LAB strains showed antibiotic resistance, antimicrobial activity against pathogens, and tolerance to gastrointestinal juices. Isolated LAB could not survive in gastric juice at pH 2, and this reduction of survival of LAB at pH 2 has previously been reported elsewhere. The viable counts of many strains of *L. brevis* and *L. plantarum* isolated from traditional Malaysian fermented Bambangan were dramatically reduced from 7.6–8 log CFU/mL to <2 log CFU/mL after exposure to phosphate buffer at pH 2 for 2 h (Ng et al., 2015). However, our findings are contradictory to those of Unban et al. (2021) who demonstrated that *Lactiplantibacillus plantarum* and *Lactiplantibacillus pentosus* isolated from Miang were able to resist simulated gastric juice at pH 2 with a survival rate > 80%. In addition, Klayraung and Okonogi (2009) found that *Limosilactobacillus fermentum* from Miang exhibited a survival rate in simulated gastric juice at pH 2 of >50%. This discrepancy may be related to the fact that the pH of the Miang sampled in this study, which was the source of the LAB analyzed, was approximately 4.

The properties of LAB are affected by their growth environment. In particular, the influence of symbiotic microorganisms is significant. In Ishizuchi-kurocha, metabolites and fermentation heat caused by *Aspergillus* are considered to be involved in the selection of LAB involved in lactate fermentation. Although *Lactiplantibacillus plantarum* isolated from Ishizuchi-kurocha is highly resistant to penicillin, the penicillin tolerance of *Lactiplantibacillus pentosus* isolated from Awa-bancha, which does not include fungal fermentation, was lower (Horie et al., 2019a). However, many LAB strains isolated from Miang exhibited broad-spectrum antibiotic resistance. This antibiotic resistance may involve metabolites from the fungal flora of Miang. Moreover, the LAB isolated from Miang exhibited antibacterial activity. A previous study also reported that *Limosilactobacillus fermentum* isolated from Miang exhibited antibacterial activity against pathogens such as *Listeria monocytogenes*, *Salmonella enterica* serovar Typhi, *Shigella sonnei*, and *Staphylococcus aureus* (Klayraung and Okonogi, 2009). These results suggest that LAB in Miang prevent the growth of pathogenic bacteria during fermentation. The production method for Miang established by our predecessors may have selected LAB with antibacterial activity. By contrast, the LAB isolated from Miang did not show antifungal activity against *Candida* spp. The lack of antifungal activity against *Candida* may be because *Candida* is the dominant species in the fungal flora of Miang and coexists with LAB.

In this study, several promising probiotic strains among the LAB isolated from Miang were identified. The properties of these strains may be closely related to the microorganisms involved in Miang fermentation. These LAB strains are expected to be used as probiotics for application within the food industry in the future. On the other hand, it remains to be determined whether the probiotic activity observed in this study is present during the actual fermentation process of tea leaves in Miang. Furthermore, it needs to be explored whether similar probiotic effects can be achieved in the food industry, such as during fermentation processes using milk as a substrate.

## 5 Conclusion

*Lactobacillaceae* bacteria were the most dominant species involved in Miang fermentation, regardless of region. At the species level, *Lactiplantibacillus pentosus* dominates, but regional diversity was observed. In this study, 2.6% of all isolates were found to have probiotic potential. In practical food applications, both functionality and taste are crucial attributes. Future research must focus on enhancing the added value and palatability of fermented foods, such as yogurt and pickles.

## Data Availability

Original dataets are available in a publicly accessible repository: The original contributions presented in the study are publicly available. This data can be found here: https://www.ncbi.nlm.nih.gov/bioproject. Accession No.:PRJDB18930.
